# Prevalence of High-Risk Human Papillomavirus Infection, Associated Risk Factors, and Relationship With Cervical Precancerous Lesions in Perimenopausal and Older Women in an Area With High Cervical Cancer Incidence in China

**DOI:** 10.7759/cureus.58081

**Published:** 2024-04-11

**Authors:** Ruoyi Zhang, Wei Xu, Siyuan Yang, Dehua Hu, Li Bai, Rumei Xiang, Xiaowei Zhao, Yuxian Nie, Qiu-ling Shi

**Affiliations:** 1 Epidemiology and Health Statistics, School of Public Health, Chongqing Medical University, Chongqing, CHN; 2 Nursing, School of Nursing, Chongqing Medical University, Chongqing, CHN; 3 Obstetrics and Gynecology, Centre of Lueyang Maternal and Child Health Care Hospital, Shaanxi, CHN; 4 Epidemiology and Health Statistics, College of Public Health, Chongqing Medical University, Chongqing, CHN; 5 Biomedical Engineering, State Key Laboratory of Ultrasound in Medicine and Engineering, Chongqing Medical University, Chongqing, CHN; 6 Epidemiology and Health Statistics, College of Public Health, State Key Laboratory of Ultrasound in Medicine and Engineering, Chongqing Medical University, Chongqing, CHN

**Keywords:** influencing factors, precancerous lesions, older women, perimenopausal women, cervical cancer, hr-hpv infection

## Abstract

Purpose

This study delves into the epidemiology of high-risk human papillomavirus (HR-HPV) infection and its link to precancerous lesions among perimenopausal (40-59 years) and elderly (60-65 years) women in a Chinese county with a notably high incidence of cervical cancer. By uniquely focusing on these age groups in underdeveloped regions, the research aims to offer novel strategies for the management and prevention of cervical cancer. It seeks to inform targeted interventions and public health policies that could significantly benefit women at heightened risk for HPV, addressing a critical gap in current prevention efforts in economically disadvantaged communities.

Methods

This observational study was conducted at the Maternal and Child Health and Family Planning Service Centre in Lueyang County, from September 2021 to January 2022. It assessed 2008 women aged 40-65 for HPV screening, with 342 undergoing further cytological examination. The study evaluated the prevalence of HPV infection across different age groups and risk categories. It utilized a questionnaire to collect participants' basic information, health behaviors, and other relevant data to analyze factors influencing HR-HPV infection. Statistical analyses comprised chi-square tests, trend analysis, logistic regression, and multiple imputation techniques to address missing data.

Results

The prevalence of HR-HPV infection among women aged 40-65 years in Lueyang County was 18.43%. Older women exhibited a higher incidence of HPV infection, abnormal ThinPrep Cytology Test (TCT) results (Shaanxi Fu'an Biotechnology Co. Ltd., Baoji City, China), and low/high-grade squamous intraepithelial lesions (LSIL/HSIL) (P<0.05). The most prevalent HR-HPV genotypes in the overall, perimenopausal, and elderly groups were HPV-52, -53, and -58; HPV-52, -53, and -16; and HPV-58, -52, and -53, respectively. The prevalent HR-HPV genotypes in the abnormal The Bethesda System (TBS) results were HPV-16, -52, -33, -58; -16, -52, -58; and-16, -33, and -52. HPV-16, -18, -33 prevalence increased with increasing lesion severity (P<0.05). In this study, factors affecting HR-HPV in the three age groups were found to be mainly related to sexual behavior and education level, including history of lower genital tract diseases, multiple pregnancies, contraceptive methods without tubal ligation, age at first marriage greater than 18 years, never washing the vulva after sex, abstinence from sex, education level of junior high school or above, and spouse's education level of high school or above.

Conclusions

These findings suggest that the elevated rate of abnormal TBS in the older age group may be attributed to the higher prevalence of persistent infection-prone HR-HPV genotypes (HPV-58, -52, and-53), multiple infections, and potent oncogenic HR-HPV genotypes (HPV-16 and -33). Additionally, the higher HR-HPV prevalence in older patients may be related to lower education attainment, reduced screening rate, and limited condom usage. Therefore, strategies targeting perimenopausal and older women should prioritize enhancing health awareness, increasing screening rates, and encouraging condom utilization.

## Introduction

Cervical cancer is the fourth most common cancer diagnosed globally. In 2020, it was estimated that there were approximately 604,000 new cases, leading to 342,000 fatalities [1]. The incidence of cervical cancer varies by region and age group. In more affluent countries, incidence peaks at around 40 years of age. However, in less economically developed countries, the incidence continues to increase until the age of 55-69 years [2]. A strong association exists between cervical cancer and persistent infection with high-risk human papillomavirus (HR-HPV), with the risk of persistence increasing with age [3].

In China, individuals aged 60 years and over represent 23.5% of the total cervical cancer incidence rate [4], with the highest rate of squamous cell carcinoma seen in those aged 50 years and over [5]. With the global population aging rapidly, the number of older women is increasing every day [6]. Older women diagnosed with cervical cancer have a higher risk of mortality within three years compared to younger women [7]. They are also more likely to choose primary radiotherapy or to forgo treatment entirely [8]. Thus, older women represent a significant public health challenge in achieving the World Health Organization's global strategy for the elimination of cervical cancer [2].

Prior research has highlighted the dominance of certain HR-HPV genotypes, such as HPV-16 and HPV-18, in the etiology of cervical cancer and underscored the importance of early detection and treatment of high-grade precancerous lesions to reduce cancer incidence [9]. However, the epidemiology of HR-HPV infections and the link between specific genotypes and the development of precancerous lesions in older women have received less attention. Although the incidence of cervical cancer is declining in China [10], Lueyang County in Shaanxi Province continues to report one of the highest cervical cancer rates, while its average income remains one of the lowest in the country [11]. This underscores the need for focused research in areas like Lueyang County, where cervical cancer incidence is high and economic levels are low.

This study aims to address these gaps by exploring the epidemiology and factors influencing HR-HPV infection and the relationship between HR-HPV genotypes and precancerous lesions among perimenopausal and older women in Lueyang County. By shedding light on these associations, the study seeks to contribute valuable insights towards the development and refinement of a standardized, holistic management model for HR-HPV infections and cervical-related diseases in the region, ultimately aiming to reduce morbidity and mortality associated with cervical cancer.

This article was previously published on 20 September 2023 on the Research Square preprint server.

## Materials and methods

Study participants

This study investigated all women who received HPV screening between September 2021 and January 2022 at the Maternal and Child Health and Family Planning Service Center in Lueyang County, Shaanxi Province. Inclusion criteria included individuals: (1) aged between 40 and 65 years, (2) residents of Lue Yang County for at least six months, (3) having engaged in sexual intercourse, (4) abstaining from sexual intercourse, vaginal douching, drug applications, and any form of transvaginal manipulation for three days prior to the examination, and (5) capable of fully understanding the study, signing the informed consent form, and voluntarily participating. Exclusion criteria included individuals: (1) who were pregnant or menstruating,(2) with a history of hysterectomy or cervical resection,(3) who had cognitive disorders preventing them from understanding or completing the study, or who refused to cooperate with the investigation.

Specimen collection

Before the examination, study participants were instructed to abstain from sexual intercourse, vaginal medications, and douching and should not have had menstruated in the last three days before the examination. Experienced gynecologists collected cervical and vaginal cell samples from the cervical canal using cell brushes. The collected samples were submerged in a fixative solution or coated on fixed pieces. Subsequently, they were stored in a specimen transport medium. To maintain temperature stability between 2-8℃, the specimens were stored in an incubator with ice. Subsequently, all specimens were transported within 24 h to a medical laboratory in Xi'an, Shaanxi Province, for HPV DNA genotyping.

DNA extraction and HPV genotyping

DNA was extracted using Yaneng Biotechnology's HPV Nucleic Acid Extraction Reagent (Yaneng Biotechnology, Shenzhen, China). The method followed the manufacturer's protocol for cervical and vaginal cell samples. Polymerase chain reaction (PCR)-reverse dot blot hybridization was utilized to detect 23 HPV subtypes. The panel included 17 HR-HPV types: HPV-16, -18, -31, -33, -35, -39, -45, -51, -52, -53, -56, -58, -59, -66, -68, -73, and -82. Additionally, six low-risk human papillomavirus (LR-HPV) genotypes were identified: HPV-6, -11, -42, -43, -81, and -83 [12].

ThinPrep cytologic test

In this study, samples were obtained from the squamo-columnar junction, also known as the transformation zone of the cervix, using a specialized cervical cytology brush for meticulous examination of the cervical canal. Utmost precautions were taken to prevent any cervical injury, which could lead to bleeding and potentially compromise the results. For the liquid-based cytology method, the ThinPrep cytologic test (Shaanxi Fu'an Biotechnology Co. Ltd., Baoji City, China) was used. The collected cells were either immediately washed or the brush head was directly placed into a vial containing preservation solution, preparing the samples for subsequent analysis.

HPV genotype categories

We aimed to analyze HPV infection rates in different categories as follows comprehensively: (1) overall and genotype-specific infection rates; (2) HR-HPV, LR-HPV, and mixed-risk HPV categories (mixed high-risk and low-risk HPV infections), based on human carcinogenic factor classification; (3) single, dual, and multiple HR-HPV infection groups (infected with three or more genotypes); (4) age-related prevalence, with participants grouped into five categories: 40-44, 45-49, 50-54, 55-59, and 60-65 years; and (5) the perimenopausal (40-59 years) and older groups (60-65 years).

Questionnaire design

The questionnaire design was influenced by a comprehensive review of domestic and international literature [13-15]. We incorporated professional expertise and sought consultation from gynecologists, ensuring the questionnaire aligned with the specific characteristics of Lueyang County. The questionnaire included basic information, menstrual history, marital history, reproductive history, personal medical history, family oncological history, sexual behavior, hygienic behavior, cognitive situation, basic information about spouse or sexual partner, marital history, and circumcision history.

The investigators were uniformly trained, standardized terminology was unified, and face-to-face conversations were used to conduct the survey, and the study participants and managing doctors were required to sign the Informed Consent Form.

Ethics approval

Ethical approval for this study was granted by the Maternity Service Center of Lueyang Maternal and Child Health Hospital on December 4, 2021 (Approval No. 2021-001).

Statistical analysis

Analyses were performed using specific software per test type: Chi-square tests and trend analyses with IBM SPSS (version 23.0, IBM Corp., Armonk, USA), logistic regression models in SAS (version 9.4), and the handling of missing data through multiple imputation techniques in R (version 4.2.1, R Foundation for Statistical Computing, Vienna, Austria).

Count data rates (%) were analyzed using the Chi-square (χ2) test, which demonstrated the prevalence of specific types of HPV and cervical lesions. The Cochran-Armitage trend test was used to assess whether there was a linear relationship between the HR-HPV infection rate, histological findings, and age.

Univariate and multivariate logistic regression analyses were performed to investigate the correlation between HR-HPV positivity and risk factors obtained from the questionnaire. An inverse stepwise likelihood ratio test was used for the multivariate logistic regression analysis to explore the factors influencing HR-HPV. Additionally, the Hosmer-Lemeshow test was employed to evaluate model fit. Risk factors with a p<0.10 in the univariate logistic regression analysis were included in the risk regression analysis. The statistical significance level was set at P<0.05.

To address the missing data, the mice package, and Visualization and Imputation of Missing Values (VIM)** **package of the R software was utilized to explore the proportion and type of missing data. The pattern of missing data was determined through a correlation matrix known as the shadow matrix. Multiple imputation techniques were employed to handle the missing data and subsequently evaluated the reliability of the interpolated results. Interpolated results with the highest Cronbach's alpha coefficients were used for the multifactor analysis following multiple imputations.

## Results

Age-specific prevalence of HPV infection

**Figure 1:**
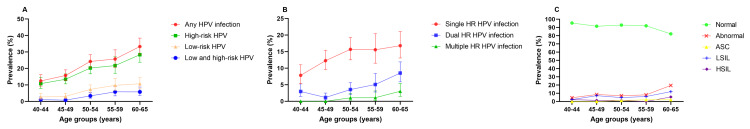
Prevalence of HPV infection and TBS results by age group. A: Any, high-risk, low-risk, and mixed-risk HPV infections; B: Single, dual, and multiple HR-HPV infections; C: TBS diagnostic results. HPV: Human Papillomavirus; TBS: The Bethesda System; HR-HPV: High-risk Human Papillomavirus; ASC: Atypical Squamous Cells; LSIL: Low-grade Squamous Intraepithelial Lesion; HSIL: High-grade Squamous Intraepithelial Lesion.

Overall, 2,008 women aged 40-65 years were enrolled in this study, with an overall HPV prevalence of 21.71% (95% CI 19.93%-23.58%). Specifically, the prevalence rates for HR-HPV, Low-risk human papillomavirus (LR-HPV), and mixed-risk HPV infections were 18.43%, 6.32%, and 3.04%, respectively. The prevalence of HPV, HR-HPV, and LR-HPV infections increased with age, peaking at 60-65 years (HPV, 33.30%; HR-HPV, 28.37%; and LR-HPV, 10.74%). The prevalence of HPV, HR-HPV, LR-HPV, and mixed-risk HPV infections was significantly higher in the older group than in the perimenopausal group (P<0.001). The Cochran-Armitage trend test revealed a clear linear trend in the prevalence of HR-HPV infection with age (P<0.001) (Figure 1, Table 1).

**Table 1:** Prevalence of HPV infection grouped by age group and genotype. a Percentage of all women in study; b Percentage of women in each group; c The perimenopausal group compared with the elderly group; HPV: Human Papillomavirus; HR-HPV: High-risk Human Papillomavirus; LR-HPV: Low-risk Human Papillomavirus.

HPV genotype	n(%^a^) (N=2008)	95% CI (%^a^)	40-44(%^b^) (n=370)	45-49(%^b^) (n=521)	50-54(%^b^) (n=478)	55-59(%^b^) (n=276)	40-59(%^b^) Premenopausal group（n=1645）	60-65(%^b^) Elderly group (n=363)	χ2^(c)^	P-Value ^(c)^
Type of infection										
HPV	436(21.70)	19.93-23.58	46(12.43)	82(15.74)	116(24.20)	71(25.72)	315(19.15)	121(33.30)	35.198	<0.001
HR-HPV	370(18.40)	16.75-20.19	40(10.81)	70(13.44)	97(20.29)	60(21.74)	267(16.23)	103(28.37)	29.176	<0.001
LR-HPV	127(6.32)	5.30-7.48	10(2.70)	16(3.07)	35(7.32)	27(9.78)	88(5.35)	39(10.74)	14.605	<0.001
Mixed-risk HPV	61(3.04)	2.30-3.89	4(1.08)	4(0.77)	16(3.35)	16(5.80)	40(2.43)	21(5.79)	11.354	0.001
Number of HR-HPV infection										
1	272(13.55)	12.08-15.12	29(7.84)	64(12.28)	75(15.69)	43(15.58)	211(12.83)	61(16.80)	4.018	0.045
2	79(3.93)	3.13-4.88	11(2.97)	6(1.15)	17(3.56)	14(5.07)	48(2.92)	31(8.54)	24.869	<0.001
≥3	19(0.95)	0.57-1.47	0(0.00)	0(0.00)	5(1.05)	3(1.09)	8(0.49)	11(3.03)	-	<0.001(Fisher)
HR HPV										
HPV-16	53(2.64)	1.98-3.44	6(1.62)	10(1.92)	12(2.51)	11(3.99)	39(2.37)	14(3.86)	2.555	0.110
HPV-18	25(1.25)	0.81-1.83	3(0.81)	5(0.96)	5(1.05)	6(2.17)	19(1.16)	6(1.65)	-	0.433（Fisher）
HPV-31	22(1.1)	0.69-1.65	1(0.27)	3(0.58)	6(1.26)	1(0.36)	11(0.67)	11(3.03)	-	0.001(Fisher)
HPV-33	29(1.44)	0.97-2.07	1(0.27)	6(1.15)	6(1.26)	5(1.81)	18(1.09)	11(3.03)	7.831	0.005
HPV-35	9(0.45)	0.21-0.85	2(0.54)	1(0.19)	3(0.63)	1(0.36)	7(0.43)	2(0.55)	-	0.670(Fisher)
HPV-39	18(0.9)	0.53-1.41	2(0.54)	4(0.77)	3(0.63)	2(0.72)	11(0.67)	7(1.93)	-	0.031(Fisher)
HPV-45	7(0.35)	0.14-0.72	1(0.27)	0(0.00)	3(0.63)	1(0.36)	5(0.30)	2(0.55)	-	0.617(Fisher)
HPV-51	32(1.59)	1.09-2.24	5(1.35)	4(0.77)	7(1.46)	4(1.45)	20(1.22)	12(3.31)	8.283	0.004
HPV-52	76(3.78)	2.99-4.71	6(1.62)	12(2.3)	25(5.23)	12(4.35)	55(3.34)	21(5.79)	4.868	0.027
HPV-53	57(2.84)	2.16-3.66	10(2.7)	6(1.15)	16(3.35)	9(3.26)	41(2.49)	16(4.41)	3.955	0.047
HPV-56	31(1.54)	1.05-2.18	4(1.08)	6(1.15)	7(1.46)	7(2.54)	24(1.46)	7(1.93)	0.431	0.512
HPV-58	55(2.74)	2.07-3.55	3(0.81)	7(1.34)	14(2.93)	7(2.54)	31(1.88)	24(6.61)	24.943	<0.001
HPV-59	23(1.15)	0.73-1.71	2(0.54)	5(0.96)	8(1.67)	4(1.45)	19(1.16)	4(1.10)	-	1.000(Fisher)
HPV-66	25(1.25)	0.81-1.83	2(0.54)	1(0.19)	5(1.05)	4(1.45)	12(0.73)	13(3.58)	-	<0.001(Fisher)
HPV-68	23(1.15)	0.73-1.71	2(0.54)	5(0.96)	4(0.84)	5(1.81)	16(0.97)	7(1.93)	-	0.166(Fisher)
HPV-73	5(0.25)	0.08-0.58	1(0.27)	1(0.19)	0(0.00)	1(0.36)	3(0.18)	2(0.55)	-	0.224(Fisher)
HPV-82	4(0.2)	0.05-0.51	0(0.00)	0(0.00)	0(0.00)	0(0.00)	0(0.00)	4(1.10)	-	0.001(Fisher)
LR HPV										
HPV-6	11(0.55)	0.27-0.98	0(0.00)	1(0.19)	3(0.63)	3(1.09)	7(0.43)	4(1.10)	-	0.121(Fisher)
HPV-11	2(0.1)	0.01-0.36	0(0.00)	0(0.00)	1(0.21)	0(0.00)	1(0.06)	1(0.28)	-	0.329(Fisher)
HPV-42	67(3.34)	2.60-4.22	3(0.81)	7(1.34)	18(3.77)	13(4.71)	41(2.49)	26(7.16)	20.109	<0.001
HPV-43	22(1.1)	0.69-1.65	1(0.27)	6(1.15)	6(1.26)	5(1.81)	18(1.09)	4(1.10)	-	1.000(Fisher)
HPV-81	40(1.99)	1.43-2.70	6(1.62)	5(0.96)	12(2.51)	8(2.9)	31(1.88)	9(2.48)	0.539	0.463
HPV-83	3(0.15)	0.03-0.44	0(0.00)	0(0.00)	1(0.21)	1(0.36)	2(0.12)	1(0.28)	-	0.450(Fisher)

The prevalence rates for single, dual, and multiple HR-HPV infections were 13.55% (95% CI 12.08%-15.12%), 3.93% (95% CI 3.13%-4.88%), and 0.95% (95% CI 0.57%-1.47%), respectively. The prevalence of single (P=0.045), double (P< 0.001), and multiple HR-HPV (P<0.001) infections was significantly higher in the older age group than in the perimenopausal group (Figure 1, Table 1).

Prevalence of HPV infection by genotype groups

**Figure 2:**
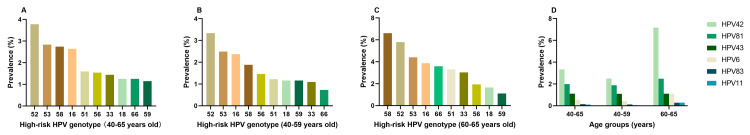
HR-HPV and LR-HPV genotype prevalence in three age groups. HPV: Human Papillomavirus; HR-HPV: High-risk human papillomavirus; LR-HPV: low-risk human papillomavirus.

The top three HR-HPV genotypes with the highest infection rates in the overall, perimenopausal, and elderly groups were as follows: HPV-52 (3.78%), -53 (2.84%), and -58 (2.74%); HPV-52 (3.34%), -53 (2.49%), and -16 (2.37%); and HPV-58 (6.61%), -52 (5.79%), and -53 (4.41%), respectively (Figure 2). Moreover, the infection rates of HPV-31, -33, -39, -42, -51, -53, -58, -66, and -82 were notably higher in the elderly group than in the perimenopausal group (P<0.05).

HR-HPV genotype distribution and number of HR-HPV infections across different TBS diagnostic outcomes and age groups

Overall, 342 individuals underwent thin-layer cytological testing, and the prevalence of HR-HPV infection was 89.77% (n=307). Based on the The Bethesda System (TBS) diagnosis, participants were divided into four groups: normal (n=306), atypical squamous cells (ASC) (ASC-US (n=3); ASC-H (n=1)), low-grade squamous intraepithelial lesion (LSIL) (n=24), and high-grade squamous intraepithelial lesion (HSIL) groups (n=8).

The age group with the highest incidence of abnormal TBS was 60-65 years (19.57%), while the age group with the lowest incidence was 40-44 years (4.55%). The incidence of ASC, LSIL, and HSIL peaked in the age group 60-65 years (ASC: 2.17%; LSIL: 11.96%; HSIL: 5.43%), while the lowest values were in the age group 40-49 years (0%), 40-44 years (2.27%), and 55-59 years (0%) (Figure 1, Table 2). Significant differences were observed in the prevalence of abnormal (P<0.001), LSIL (P=0.030), and HSIL (P=0.035) between the perimenopausal and older age groups. The Cochran-Armitage trend test revealed a linear trend between abnormalities and age (P=0.005).

**Table 2:** Distribution of TBS diagnostic results among different age groups. a The perimenopausal group compared with the elderly group; b Cochran-Armitage trend test of TBS results with age. TBS: The Bethesda System; ASC: Atypical Squamous Cells; LSIL: Low-grade Squamous Intraepithelial Lesion; HSIL: High-grade Squamous Intraepithelial Lesion.

Pathological type	40-44 n (%) (n=44)	45-49 n (%) (n=71)	50-54 n (%) (n=85)	55-59 n (%) (n=50)	40-59 n (%) （n=250）	60-65 n (%) (n=92)	χ2^(a)^	P-Value^ (a)^	χ2^(b)^	P-Value ^(b)^
Normal	42(95.45)	65(91.55)	79(92.94)	46(92.00)	232(92.80)	74(82.22)	10.918	<0.001	8.000	0.005
Abnormal	2(4.55)	6(8.45)	6(7.06)	4(8.00)	18(7.20)	18(19.57)	10.918	<0.001	8.000	0.005
ASC	0(0.00)	0(0.00)	1(1.18)	1(2.00)	2(0.80)	2(2.17)	-	0.294	2.267	0.132
LSIL	1(2.27)	5(7.04)	4(4.71)	3(6.00)	13(5.20)	11(11.96)	4.705	0.030	3.833	0.050
HSIL	1(2.27)	1(1.41)	1(1.18)	0(0.00)	3(1.20)	5(5.43)	-	0.035	1.857	0.173

The prevalence of HR-HPV infection in patients with abnormal TBS results (ASC, LSIL, and HSIL) was 100%. Additionally, the prevalence and number of infections for each HR-HPV genotype did not differ significantly between the older and perimenopausal groups. The three most common HR-HPV genotypes among patients with abnormal TBS outcomes in the overall, perimenopausal, and older groups were HPV-16 (33.33%), -52 (22.22%), -33 (19.44%), and -58 (19.44%); HPV-16 (38.89%), -52 (22.22%), and -58 (22.22%); and HPV-16 (27.78%), -33 (22.22%), and -52 (22.22%) (Table 3).

**Table 3:** Distribution of HR-HPV genotypes and number of HR-HPV infections in different TBS diagnostic results. a The perimenopausal group compared with the elderly group; b Comparison between different TBS results in the overall group; c Comparison between different TBS outcomes in the perimenopausal group; d Comparison between different TBS outcomes in the elderly group; HPV: Human Papillomavirus; HR-HPV: High-risk Human Papillomavirus; TBS: The Bethesda System; ASC: Atypical Squamous Cells; LSIL: Low-grade Squamous Intraepithelial Lesion; HSIL: High-grade Squamous Intraepithelial Lesion.

HR-HPV Genotypes	Normal				Abnormal				ASC				LSIL				HSIL				40-65	40-59	60-65
	40-65 n(%) (n=306)	40-59 n(%) (n=232)	60-65 n(%) (n=74)	P-Value ^(a)^	40-65 n(%) (n=36)	40-59 n(%) (n=18)	60-65 n(%) (n=18)	P-Value^(a)^	40-65 n(%) (n=4)	40-59 n(%) (n=2)	60-65 n(%) (n=2)	P-Value ^(a)^	40-65 n(%) (n=24)	40-59 n(%) (n=13)	60-65 n(%) (n=11)	P-Value ^(a)^	40-65 n(%) (n=8)	40-59 n(%) (n=3)	60-65 n(%) (n=5)	P-Value ^(a)^	P-Value^(b)^	P-Value^(c)^	P-Value^(d)^
HPV-16	2(0.65)	0(0.00)	2(2.7)	0.058	12(33.33)	7(38.89)	5(27.78)	0.480	0(0.00)	0(0.00)	0(0.00)	-	7(29.17)	5(38.46)	2(18.18)	0.386	5(62.5)	2(66.67)	3(60)	1.000	<0.001	<0.001	<0.001
HPV-18	3(0.98)	2(0.86)	1(1.35)	0.566	4(11.11)	1(5.56)	3(16.67)	0.603	0(0.00)	0(0.00)	0(0.00)	-	3(12.5)	1(7.69)	2(18.18)	0.576	1(12.5)	0(0.00)	1(20)	1.000	0.004	0.202	0.024
HPV-31	19(6.21)	10(4.31)	9(12.16)	0.024	3(8.33)	1(5.56)	2(11.11)	1.000	0(0.00)	0(0.00)	0(0.00)	-	2(8.33)	1(7.69)	1(9.09)	1.000	1(12.5)	0(0.00)	1(20)	1.000	0.562	0.568	0.853
HPV-33	19(6.21)	14(6.03)	5(6.76)	0.786	7(19.44)	3(16.67)	4(22.22)	1.000	0(0.00)	0(0.00)	0(0.00)	-	5(20.83)	3(23.08)	2(18.18)	1.000	2(25)	0(0.00)	2(40)	0.464	0.019	0.128	0.066
HPV-35	9(2.94)	7(3.02)	2(2.7)	1.000	0(0.00)	0(0.00)	0(0.00)	-	0(0.00)	0(0.00)	0(0.00)	-	0(0.00)	0(0.00)	0(0.00)	-	0(0.00)	0(0.00)	0(0.00)	-	1.000	1.000	1.000
HPV-39	16(5.23)	10(4.31)	6(8.11)	0.230	1(2.78)	0(0.00)	1(5.56)	1.000	1(25)	0(0.00)	1(50)	1.000	0(0.00)	0(0.00)	0(0.00)	-	0(0.00)	0(0.00)	0(0.00)	-	0.224	1.000	0.254
HPV-45	6(1.96)	5(2.16)	1(1.35)	1.000	1(2.78)	0(0.00)	1(5.56)	1.000	0(0.00)	0(0.00)	0(0.00)	-	1(4.17)	0(0.00)	1(9.09)	0.458	0(0.00)	0(0.00)	0(0.00)	-	0.544	1.000	0.355
HPV-51	29(9.48)	19(8.19)	10(13.51)	0.173	2(5.56)	0(0.00)	2(11.11)	0.486	0(0.00)	0(0.00)	0(0.00)	-	2(8.33)	0(0.00)	2(18.18)	0.199	0(0.00)	0(0.00)	0(0.00)	-	1.000	1.000	0.873
HPV-52	64(20.92)	50(21.55)	14(18.92)	0.628	8(22.22)	4(22.22)	4(22.22)	1.000	1(25)	1(50)	0(0.00)	1.000	6(25)	3(23.08)	3(27.27)	1.000	1(12.5)	0(0.00)	1(20)	1.000	0.848	0.620	0.915
HPV-53	48(15.69)	34(14.66)	14(18.92)	0.380	5(13.89)	3(16.67)	2(11.11)	1.000	0(0.00)	0(0.00)	0(0.00)	-	3(12.5)	2(15.38)	1(9.09)	1.000	2(25)	1(33.33)	1(20)	1.000	0.787	0.655	0.909
HPV-56	28(9.15)	22(9.48)	6(8.11)	0.721	2(5.56)	1(5.56)	1(5.56)	1.000	1(25)	1(50)	0(0.00)	1.000	1(4.17)	0(0.00)	1(9.09)	0.458	0(0.00)	0(0.00)	0(0.00)	-	0.489	0.180	1.000
HPV-58	45(14.71)	24(10.34)	21(28.38)	<0.001	7(19.44)	4(22.22)	3(16.67)	1.000	0(0.00)	0(0.00)	0(0.00)	-	4(16.67)	3(23.08)	1(9.09)	0.596	3(37.5)	1(33.33)	2(40)	1.000	0.293	0.181	0.410
HPV-59	20(6.54)	16(6.9)	4(5.41)	0.791	0(0.00)	0(0.00)	0(0.00)	-	0(0.00)	0(0.00)	0(0.00)	-	0(0.00)	0(0.00)	0(0.00)	-	0(0.00)	0(0.00)	0(0.00)	-	0.703	1.000	1.000
HPV-66	20(6.54)	12(5.17)	8(10.81)	0.105	3(8.33)	0(0.00)	3(16.67)	0.229	0(0.00)	0(0.00)	0(0.00)	-	3(12.5)	0(0.00)	3(27.27)	0.082	0(0.00)	0(0.00)	0(0.00)	-	0.514	1.000	0.405
HPV-68	20(6.54)	15(6.47)	5(6.76)	1.000	2(5.56)	0(0.00)	2(11.11)	0.486	1(25)	0(0.00)	1(50)	1.000	1(4.17)	0(0.00)	1(9.09)	0.458	0(0.00)	0(0.00)	0(0.00)	-	0.379	1.000	0.212
HPV-73	4(1.31)	2(0.86)	2(2.7)	0.247	0(0.00)	0(0.00)	0(0.00)	-	0(0.00)	0(0.00)	0(0.00)	-	0(0.00)	0(0.00)	0(0.00)	-	0(0.00)	0(0.00)	0(0.00)	-	1.000	1.000	1.000
HPV-82	3(0.98)	0(0.00)	3(4.05)	0.014	1(2.78)	0(0.00)	1(5.56)	1.000	0(0.00)	0(0.00)	0(0.00)	-	1(4.17)	0(0.00)	1(9.09)	0.458	0(0.00)	0(0.00)	0(0.00)	-	0.360	1.000	0.588
HR-HPV infection																							
Yes	306(88.56)	232(84.91)	74(100)	<0.001	36(100)	18(100)	18(100)	-	4(100)	2(100)	2(100)	-	24(100)	13(100)	11(100)	-	8(100)	3(100)	5(100)	-	0.342	0.681	-
No	35(11.44)	35(15.09)	0(0.00)	-	0(0.00)	0(0.00)	0(0.00)	-	0(0.00)	0(0.00)	0(0.00)	-	0(0.00)	0(0.00)	0(0.00)	-	0(0.00)	0(0.00)	0(0.00)	-	-	-	
Number of HR-HPV infection																							
0	35(11.44)	35(15.09)	0(0.00)	<0.001	0(0.00)	0(0.00)	0(0.00)	-	0(0.00)	0(0.00)	0(0.00)	-	0(0.00)	0(0.00)	0(0.00)	-	0(0.00)	0(0.00)	0(0.00)	-	0.262	1.000	-
1	204(66.67)	159(68.53)	45(60.81)	0.220	22(61.11)	13(72.22)	9(50.00)	0.172	4(100)	2(100)	2(100)	-	15(62.5)	9(69.23)	6(54.55)	0.675	3(37.5)	2(66.67)	1(20)	0.464	0.172	1.000	0.194
2	55(17.97)	31(13.36)	24(32.43)	<0.001	7(19.44)	4(22.22)	3(16.67)	1.000	0(0.00)	0(0.00)	0(0.00)	-	4(16.67)	3(23.08)	1(9.09)	0.596	3(37.5)	1(33.33)	2(40)	1.000	0.426	0.345	0.338
≥3	12(3.92)	7(3.02)	5(6.76)	0.171	7(19.44)	1(5.56)	6(33.33)	0.088	0(0.00)	0(0.00)	0(0.00)	-	5(20.83)	1(7.69)	4(36.36)	0.142	2(25)	0(0.00)	2(40)	0.464	0.003	0.455	0.008

Significant variations in infection rates for specific HR-HPV genotypes were observed between groups with different TBS outcomes (normal, ASC, LSIL, and HSIL) in the overall, perimenopausal, and elderly groups. The overall group, HPV-16 (P<0.001), -18 (P=0.004), -33 (P=0.019), and multiple HR-HPV (P=0.003) showed varying infection rates. In the perimenopausal group, HPV-16 (P<0.001) exhibited a significant difference, while in the elderly group, HPV-16 (P<0.001), -18 (P=0.024), and multiple HR-HPV infections (P=0.008) differ significantly (Table 3).

Univariate logistic regression analysis of factors influencing HR-HPV infection

This was conducted to identify the factors influencing HR-HPV infection in three different age groups. A total of 60 variables were included in the analysis, and we identified 26 (e.g. Age (years), Profession, and Educational level, etc.), 26 (e.g. Profession, Educational level, and Native place, etc.) and 8 (e.g. Educational level, Living environment and living conditions and Age of menarche, etc.) P<0.10 factors for the overall, perimenopausal, and older age groups, respectively with P<0.10 (Tables 5-10 in the Appendices).

Proportion and type of missing data

**Figure 3:**
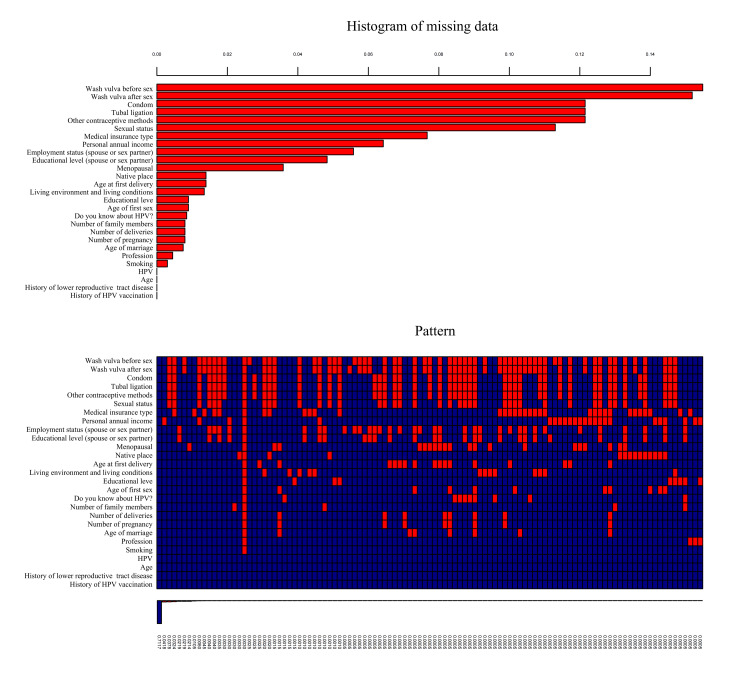
Proportion and type of missing data in the 40-65 years old group. HPV: Human Papillomavirus

**Figure 4:**
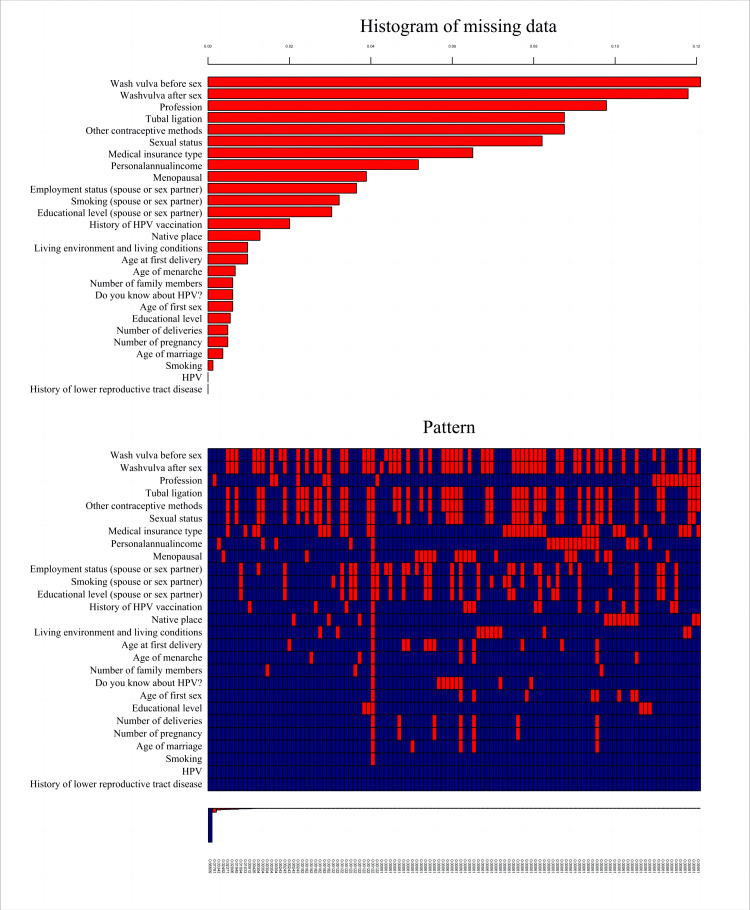
Proportion and type of missing data in the 40-59 years old group. HPV: Human Papillomavirus.

**Figure 5:**
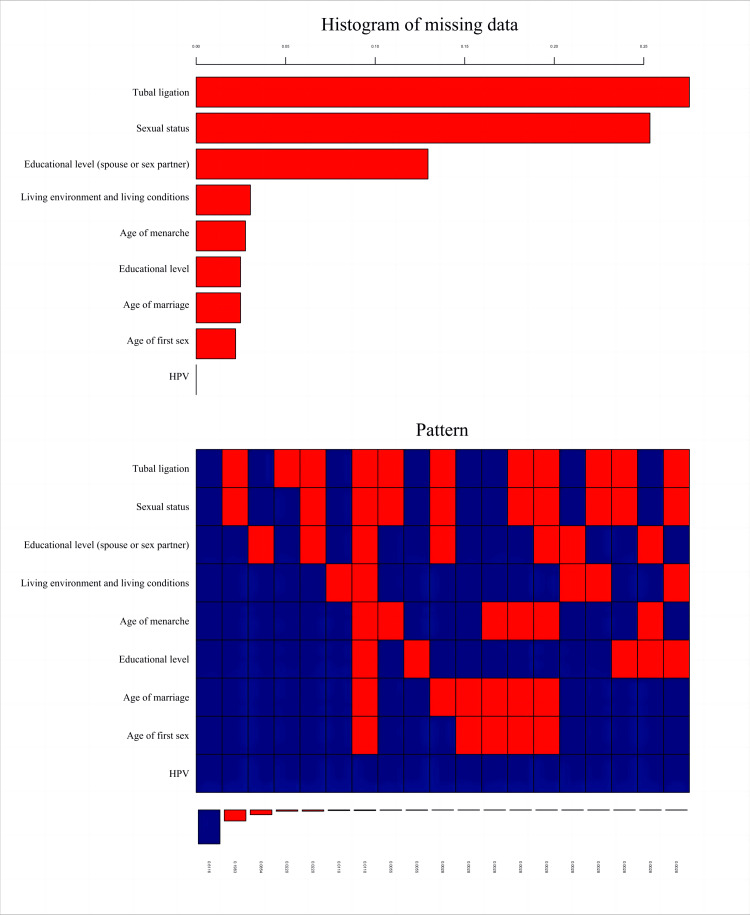
Proportion and type of missing data in the 60-65 years old group. HPV: Human Papillomavirus.

For all three groups, the missing data type was missing at random. Figure 3 illustrates the pattern of missing data for the overall group (see Figures 4-5 in the Appendices for the missing data pattern in the perimenopausal and elderly groups). The numbers below each pattern indicate the proportions of missing data for each condition. Specifically, the variables associated with sexual hygiene practices and contraceptive methods showed high rates of missing data. 

Results of multivariable logistic regression analysis following multiple interpolation

To address missing data, which were determined as missing at random, a multiple logistic regression analysis (Table 4) was conducted after multiple imputations (Table 11 in the Appendices) for each age group. The outcomes of the analysis without multiple imputations are provided in the Supplementary Material (Table 12 in the Appendices).

**Table 4:** Results of multivariate logistic regression analysis of factors affecting HR-HPV infection after multiple interpolations. HR-HPV: High-risk Human Papillomavirus.

Characteristics	classification	SE	P-Value	OR(95% CI)
The overall group				
Age	-	-	< 0.001	-
	40-44	-	-	1（Reference）
	45-49	0.218	0.565	1.13[0.74-1.74]
	50-54	0.212	<0.01	1.73[1.14-2.62]
	55-59	0.234	0.007	1.89[1.20-3.00]
	60-65	0.229	< 0.001	2.57[1.64-4.02]
Smoking	Present or past	-	-	1（Reference）
	Never	0.395	0.048	0.46[0.21-0.99]
Age of first marriage	≤18	-	-	1（Reference）
	>18	0.245	0.023	0.57[0.36-0.93]
History of lower reproductive tract disease	No	-	-	1（Reference）
	Yes	0.127	<0.01	1.39[1.08-1.78]
Number of pregnancy	-	-	0.003	-
	≤1	-	-	1（Reference）
	2	0.23	0.002	2.05[1.30-3.21]
	≥3	0.233	<0.001	2.18[1.38-3.44]
Contraceptive method: tubal ligation	Yes	-	-	1（Reference）
	No	0.157	0.011	0.67[0.49-0.91]
Educational level （Spouse or sexual partner）	Below high school	-	-	1（Reference）
	High school and above	0.147	<0.01	0.69[0.51-0.92]
The perimenopausal group				
History of lower reproductive tract disease	No	-	-	1（Reference）
	Yes	0.146	0.012	1.44[1.09-1.92]
Menopausal	Yes	-	-	1（Reference）
	No	0.145	0.005	0.66[0.50-0.88]
Number of pregnancy	-	-	0.002	-
	≤1	-	-	1（Reference）
	2	0.254	0.002	2.20[1.34-3.62]
	≥3	0.257	<0.001	2.42[1.46-4.00]
Contraceptive method: tubal ligation	Yes	-	-	1（Reference）
	No	0.181	<0.001	0.55[0.39-0.79]
Other contraceptive methods (Menopause, extracorporeal ejaculation, etc.)	Yes	-	-	1（Reference）
	No	0.287	0.003	0.42[0.24-0.74]
Wash vulva after sex	-	-	0.021	-
	Frequently	-	-	1（Reference）
	Occasionally	0.181	0.17	1.28[0.90-1.83]
	Never	0.217	<0.01	1.75[1.14-2.67]
The elderly group				
Educational level	Below junior high school	-	-	1（Reference）
	Junior high school and above	0.362	0.015	0.42[0.20-0.84]
Sexual status	Yes	-	-	1（Reference）
	No	0.321	< 0.001	0.24[0.13-0.46]
Contraceptive method: tubal ligation	Yes	-	-	1（Reference）
	No	0.381	0.006	0.35[0.17-0.74]

The factors influencing HR-HPV infection in the overall group (40-65 years) remained consistent with the results obtained without multiple imputations. These factors included a history of lower genital tract disease, multiple pregnancies, and contraception methods without tubal ligation. Additionally, we identified several new independent protective factors, including age <50 years, non-smoking status, age at first marriage >18 years, and the education level of spouse (high school or above).

The factors influencing HR-HPV infection in the perimenopausal group remained consistent with multivariate logistic regression results without multiple imputations. These factors included multiple pregnancies and contraception without tubal ligation. Additionally, we observed a history of lower genital tract disease, nonmenopausal status, avoidance of other contraceptive methods, and never cleaning their vulva after sex were independent factors.

Consistent with the multivariate logistic regression findings excluding multiple imputations, we observed that educational attainment beyond junior high school and practicing abstinence emerged as distinct protective factors against HR-HPV infection in the elderly group. Additionally, multiple imputation analyses revealed contraception without tubal ligation as an independent influencing factor. 

## Discussion

In this study, the prevalence of HR-HPV infection among women aged 40-65 years in Lueyang County was 18.43%. The prevalence of any HPV infection, abnormal TBS results, LSIL and HSIL was significantly higher in older women compared to the perimenopausal group. The top three genotypes with the highest prevalence of infection and the most common genotypes in patients with abnormal TBS results differed in the overall, perimenopausal, and older age groups. The prevalence of HPV-16, -18, -33, and multiple HR-HPV infections increased with the severity of cervical squamous intraepithelial lesions. The independent influences on HR-HPV infections were mainly related to sexual behaviors and educational attainment. 

Our study revealed that the prevalence of HR-HPV infection (18.43%) was higher than that in Western countries such as Chile (9.1%) [16], but lower than the prevalence in African countries (32.3%) [17]. In addition, the prevalence of HR-HPV infection in this study was higher than that observed in the national screening population (17.7%) [18]. However, it was comparable to that in the national rural screening population (18.0%) [18]. Based on these findings, the disparity between HR HPV infection rates in Lueyang County and the western and national average rates highlights the potential impact of socioeconomics and health awareness on HPV infection rates. Lower economic status may increase the risk of HR HPV infection by limiting access to health education and screening services [19]. In addition, lack of health awareness may lead to behaviours that increase the risk of infection, such as infrequent use of protection during sexual activity [20]. Given these observations, addressing higher rates of HR HPV infection in areas such as Lueyang County requires targeted interventions to enhance services such as health education and HPV screening.

In this study, the peaks of HPV infection and precancerous lesions were observed in older women, consistent with findings from those in less-developed countries [21]. While some studies have reported a decreased incidence of cervical intraepithelial neoplasia in older adults, they were primarily conducted in developed countries where older women are more likely to have undergone prior cervical cancer screening [2]. The elevated incidence of HPV infection and precancerous lesions in older women might be attributed to cohort effects. This cohort was born in the 1950s and 1960s, experiencing poorer economic conditions in China during their upbringing. This often resulted in lower educational levels, limited knowledge about HPV and cervical cancer, and historically low participation in cervical cancer screening [22]. Additionally, the increased HPV infection rate in older women may be associated with a higher incidence of persistent infection and lower clearance rates [23]. A study found that the correlation between the prevalence of HR-HPV infection and cervical cancer incidence increased with age [24]. The elevated rate of persistent HR-HPV infection and diminished clearance in older women might be attributed to age-related changes in the cervicovaginal epithelium and increased reactivation of latent infections due to age-related immune decline [25].

The distribution of HR-HPV genotypes varies significantly across regions worldwide [21]. In China, HPV-52, -16, and -58 are the most prevalent genotypes in multiple regions [26]. The three HR-HPV genotypes (HPV-58, -52, and -53) that exhibit the highest prevalence of infection in the elderly group in our study may be associated with their persistence, especially HPV-58, which was prominent. A retrospective cohort study conducted in Heilongjiang Province found HPV-58 and -53 as the most persistent genotypes, followed by HPV-52 and HPV-16 [27]. A study in the United States reported high type-specific persistence rates (> 30%) for HPV-31, -16, -58, -52, and -53 [28]. Additionally, a Finnish study revealed that HPV types 35, 58, and 52 exhibited the longest persistence [29]. HPV-31 and -35 are more prevalent in Europe and the United States but less common in China [30].

The most common HR-HPV genotypes found in patients with abnormal TBS results in this study were highly consistent with the regional distribution observed in Beijing (HPV-16, -52, -58, and -33) [31] and rural areas of Shanxi Province (HPV-16, -52, -33, -31, and -58) [32]. The findings of Song et al. align with those of this study, revealing a significant increase in HPV-16, -18, and -33 infection rates with a higher degree of cytologic abnormalities [33]. Multiple HR-HPV infections have been associated with an elevated risk of cervical intraepithelial neoplasia and cervical cancer [34].

Several studies have highlighted the major link between HR-HPV infection and sexual behaviour [14]. In addition, these studies have highlighted the multifactorial nature of the risk factors contributing to infection rates [15]. Among these, smoking is considered an important causative factor, not only because of its carcinogenic properties, but also because it may impair the immune response to HPV [35]. Early marriage and multiple pregnancies are also considered key risk factors for HR-HPV infection. These conditions are often associated with earlier initiation of sexual activity and a larger number of lifelong sexual partners, increasing the likelihood of exposure and infection with HR-HPV [36]. In addition, a history of lower genital tract disease, including sexually transmitted infections (STIs) other than HPV, has been associated with an increased risk of HR-HPV infection. The presence of STIs may lead to mucosal damage, facilitating HPV entry and infection. Low literacy has also been identified as an important risk factor for HR-HPV infection [37]. Limited health literacy can affect an individual's ability to access, understand, and use information related to HPV prevention and treatment, including vaccination and screening programmes. Additionally, a spouse with an educational level below high school was identified as an independent risk factor for HR-HPV infection in women, highlighting the importance of health education on HPV and cervical cancer for both sexes. Abstinence serves as a protective factor against HR-HPV infection in older individuals since sexual contact is the primary mode of transmission [12].

In our study, we discovered that tubal ligation contraception and other contraceptive methods, such as menopause and in vitro ejaculation, were independent risk factors for HR-HPV infection. Women who have undergone tubal ligation may undergo cervical screening less frequently owing to the convenience of this method, which does not necessitate regular monitoring by healthcare providers [38]. Moreover, women who have opted for these long-acting contraceptive methods are less likely to use condoms during intercourse, leading to the potential transmission of HPV through sexual activity and an increased risk of cervical damage [39]. Several studies have indicated a decline in condom use as individuals age. Although condoms are effective in preventing the transmission of sexually transmitted infections, older adults may be unaware of the associated risks and may not choose to use them [40]. In our study, the univariate logistic regression analysis revealed that condom use was associated with a reduced risk of HR-HPV infection in the overall study group, consistent with the findings of Niu et al. [15]**.** However, the multivariate logistic regression analysis showed that the protective effect of condoms was insignificant. Therefore, factors like tubal ligation and other long-acting contraception (such as during menopause) may serve as instrumental variables in decision-making regarding condom use. Thus, efforts to prevent cervical cancer among perimenopausal and older women should prioritize increasing health awareness, improving cervical cancer screening rates, and encouraging condom use [22].

However, there were some limitations to this study that were worth mentioning. Firstly, the sample size and scope might not have been fully representative of the wider population, as the data came from only one hospital in Lueyang County. This suggested that future studies would need to incorporate a more diverse and broader sample pool from multiple locations to improve representativeness. Secondly, the focus of the study was on an older age group, limited to individuals aged 60-65 years, which might have affected the generalizability of the findings to a wider group of older women. Future studies could have addressed this limitation by expanding the age range of participants to include more older women, thus providing a more comprehensive understanding of the prevalence of HR-HPV in this population and its impact.

## Conclusions

The higher rate of abnormal TBS results in older women, compared to perimenopausal women, may be attributed to factors such as a higher prevalence of HR-HPV genotypes prone to persistent infection, an increased likelihood of multiple infections, a heightened prevalence of highly oncogenic HR-HPV genotypes, lower educational attainment, lower participation in screening programs, and lower condom usage. These findings had important clinical and public health prevention implications. Specifically, the study highlighted the need for increased education about HR-HPV risk and the benefits of regular screening, as well as the need to increase access to these screenings for older women. In addition, the study highlighted the importance of promoting safe sex practices to reduce the risk of HR-HPV infection in this population. The results of the study advocated for targeted interventions that addressed the unique challenges and needs of perimenopausal and older women in order to reduce the incidence of cervical cancer in this population.

## Appendices

 Detailed process of DNA extraction and HPV genotyping

1. Cervical Cell Sample Collection Process

(1) Preparation: The vagina was opened with a speculum, and cervical mucus was gently wiped off with a cotton swab to clear the cervix area for sampling.

(2) Sampling: Holding the handle of the cell brush with the right hand, the brush's central bristles were gently inserted deep into the cervical canal. This positioning ensured the shorter bristles could fully contact the cervix. The brush was then rotated in the same direction 5-10 times to collect cells. It's crucial not to rotate the brush back and forth during sampling.

(3) Rinsing: The cell brush, now with detached cells, was placed into a vial containing cell preservation fluid for rinsing. To transfer cells into the preservation fluid effectively, the brush was repeatedly pushed to the bottom of the vial and pulled up, forcing the bristles to spread apart and release the cells, done a total of 10 times.

(4) Labeling and Documentation: The vial cap was securely tightened, and the collected individual's name and sample number were labeled on the blank tag on the vial. Personal and medical history data of the collected individual were filled out on the cytology examination request form.

Subsequently, the samples are stored in a 2-8°C specimen transport medium and transported within 24 hours to a medical laboratory in Xi'an, Shaanxi Province for HPV DNA genotyping.

2. PCR-reverse dot blot hybridization Process for Detecting HPV Subtypes

(1) Sample Preparation: Extract DNA from cervical cell samples, quantify, and assess the purity and concentration of DNA, adjusting it to the appropriate amplification concentration.

(2) PCR Amplification: Prepare the PCR reaction mixture according to the primers and reagents instructions, including a specific amount of primers, dNTPs, MgCl2, Taq DNA polymerase, and extracted DNA for each sample. Set the PCR program: initial denaturation at 95°C for 10 minutes, followed by 40 cycles of 95°C denaturation for 30 seconds, specific annealing temperature (according to primer design) for 30 seconds, 72°C extension for 60 seconds, and a final extension at 72°C for 10 minutes.

(3) Reverse Dot Blot Hybridization: Incubate the PCR product with a membrane or microarray chip pre-fixed with HPV-specific probes, using a hybridization oven or shaker at the recommended temperature for an appropriate time. Perform suitable washing steps to remove non-specific binding. Add the appropriate detection reagent and develop the signal.

(4) Result Analysis: Read the signal based on the development results; each bright spot represents the presence of a specific HPV subtype. Analyze and record the presence or absence and relative abundance of each subtype.

Questionnaire on high-risk HPV infection and related influencing factors

Code: _______________

Date of creation: ______ Year ______ Month ______ Day

1、Basic Information

(1) Your name is: __________

(2) Identity card number: ______________________

(3) Contact telephone number: ______________________

(4) Residential address: ______________________

(5) Your date of birth is: ______ year ______ month ________ day

(6)Date of screening: ______ year ______ month _______day

(7) Height: ______cm, weight: ______kg

(8) Your occupation:

○Farmer ○Self-employed ○Civil servant ○Labourer ○Retiree

○Unemployed ○Other: ______                

(9) Your level of education:

○ Illiterate/no formal education ○ Elementary school and below ○ Junior high school ○ High school (junior college) ○ College ○ Bachelor's degree ○ Master's degree ○ Doctorate ○ Unknown

(10) Your place of origin: ○Liuyang County ○Other: ______                   

(11) Your ethnicity: ○Han ○Other: ________

(12) Your religion: ○No ○Yes          

(13) Number of family members: ________

(14) Personal annual income (CNY):

○<3000 ○3000-5000 ○5000-10,000 ○10,000-30,000   

○30,000-100,000 ○>100,000 ○Don't want to disclose ○Don't know

(15) Annual household income (CNY):

○<5000 ○5000-10,000 ○10,000-30,000 ○30,000-100,000 ○>100,000 ○ Do not wish to disclose ○ Don't know

(16) Whether participate in medical insurance:

○Yes (○New Rural Cooperative Medical Care ○Urban Medical Insurance

○Other: ________)

○No

(17) Living environment and living conditions: ○Excellent ○Good ○General ○Poor   

(18) Smoking: ○No ○Quit ○Yes, not quit

(19) Alcohol consumption: ○No ○Quit ○Yes, not quit

2. Past history

(1) History of gynaecological diseases (multiple choice):

□Pelvic inflammatory disease □Adenomyosis of the uterus □Endometriosis  □Tubal abnormalities □Adnexitis □Uterine fibroids □Breast Cancer □Ovarian Cancer □Endometrial resection (ablation) □Gestational trophoblastic diseases □Odourless pregnancy □Diseases of the lower genital tract (cervix, vaginal wall, vulvar inflammation, tumours, condyloma acuminatum) □Other diseases: ________       

(2) History of blood transfusion or donation: ○ Yes ○ No

(3) History of allergy: ○Yes ○No

3、Awareness level

(1) Are you aware of HPV: ○Yes ○No

(2) Whether they have regular gynaecological examination: ○Yes ○No

(3) Do you know that HPV infection can be prevented by vaccination: ○Yes ○No

(4) Whether you have been vaccinated against HPV: ○Yes ○No

4. Menstrual history

(1) Time of menarche(years): ________

(2) Whether menopause has occurred: ○Yes (age of menopause(years): ________ ) ○No

5. Marriage and childbearing history

(1) Age at first marriage: ________ (years), number of marriages:________

(2) Age of first sexual intercourse(years): ________

(3) Whether you have given birth: ○Yes ○No

(4) Age at first delivery(years): ________

(5) Number of pregnancies: ________

(6) Number of deliveries: ○Yes ○No

(7) Number of miscarriages: ________

6、Family history

(1) Family history of tumour: ○Yes ○No

(2) Family history of cervical cancer: ○Yes ○No

7. Sexual life and hygiene

(1) Number of lifetime sexual partners: ________

(2) Have you had sex in the past year:

○No

○Yes: ○＜1 time/month ○1 time/month-4 times/month ○≥5 times/month

(3) Whether willing to use contraception:

○No

○Yes (Multiple choices allowed)

□ Intrauterine device □ In vitro contraception □ Oral contraceptive pill □ Condom □ Tubal ligation □ No contraception □ Menopause □ Others: ________           

(4) Wash the vulva before intercourse: ○Yes ○Occasionally ○No

(5) Wash the vulva after intercourse: ○Yes ○Occasionally ○No

(6) Frequency of bathing:

○1-3 days/times ○3-5 days/times ○6-7 days/times ○＞7 days/times

(7) Frequency of changing underwear:

○1-3 days/times ○4-5 days/times ○6-7 days/times ○＞7 days/times

8、Spouse (or sexual partner) information

(1) Whether you have a spouse: ○Yes ○No

(2) Literacy level of spouse:

○ Illiterate/no formal education ○Elementary school or below ○Junior high school ○High school (junior college)  ○College ○Bachelor's degree ○Master's degree ○Doctorate ○Unknown

(3) Spouse's employment status:

○Unemployed ○Part-time employment ○Full-time employment ○Student in school ○Farmer farming ○Retired ○Other _____ ○Unknown

(4) Spouse's number of marriages: _____

(5) Whether spouse has a history of circumcision: ○Yes ○No ○Unknown

(6) Whether spouse smokes: ○Yes ○No ○Unknown

(7) Whether spouse drinks alcohol: ○Yes ○No (end of question)

**Table 5:** Univariate logistic regression analysis of the overall group (40-65 years old) (P<0.1). HR-HPV: high-risk human papillomavirus; CNY: Chinese Yuan

Characteristics	Classification	No. of cases (%^a^)	No. of HR-HPV+ (%^b^)	χ2	P-value
Age (years)	40-44	370(18.43)	40(10.81)	49.933	<0.001
	45-49	521(25.95)	70(13.44)		
	50-54	478(23.8)	97(20.29)		
	55-59	276(13.75)	60(21.74)		
	60-65	363(18.08)	103(28.37)		
Profession	Farmer	1062(52.89)	243(22.88)	36.145	<0.001
	Individual business	105(5.23)	15(14.29)		
	Public servant	121(6.03)	7(5.79)		
	Worker	155(7.72)	18(11.61)		
	Retired	182(9.06)	27(14.84)		
	Unemployed	104(5.18)	16(15.38)		
	Others	270(13.45)	43(15.93)		
	Missing	9(0.45)	1(11.11)		
Educational level	Below junior high school	939(46.76)	222(23.64)	33.351	<0.001
	Junior high school and above	1051(52.34)	143(13.61)		
	Missing	18(0.90)	13(27.78)		
Native place	Lueyang	1831(91.19)	349(19.06)	3.624	0.057
	Others	149(7.42)	19(12.75)		
	Missing	28(1.39)	2(7.14)		
Number of family members	1-3	943(46.96)	155(16.44)	4.703	0.030
	≥4	1049(52.24)	212(20.21)		
	Missing	16(0.80)	3(18.75)		
Personal annual income	<10000 CNY	458(22.81)	96(20.96)	4.830	0.028
	≥10000 CNY	1421(70.77)	234(16.47)		
	Missing	129(6.42)	40(31.01)		
Medical insurance type	New rural cooperative medical care	1236(61.55)	270(21.84)	24.922	<0.001
	Urban medical insurance	516(25.7)	65(12.60)		
	Others	102(5.08)	11(10.78)		
	Missing	154(7.67)	24(15.58)		
Living environment and living conditions	Excellent or good	1352(67.33)	215(15.9)	14.759	<0.001
	General or poor	629(31.32)	145(23.05)		
	Missing	27(1.34)	10(37.04)		
Smoking	Present or past	28(1.39)	11(35.48)	8.214	0.004
	Never	1974(98.31)	358(18.14)		
	Missing	6(0.30)	1(16.67)		
History of lower reproductive tract disease	No	1407(70.07)	238(16.92)	7.139	0.008
	Yes	601(29.93)	132(21.96)		
History of hpv vaccination	Yes	20(1.00)	0(0.00)	-	0.037(Fisher)
	No	1942(96.71)	364(18.74)		
	Missing	46(2.29)	6(13.04)		
Do you know about HPV?	Yes	488(24.3)	66(13.52)	9.981	0.002
	No	1503(74.85)	299(19.89)		
	Missing	17(0.85)	5(29.41)		
Menopausal	Yes	1103(54.93)	251(22.76)	31.752	<0.001
	No	833(41.48)	106(12.73)		
	Missing	72(3.59)	13(18.06)		
Age of first marriage	≤18	86(4.28)	29(33.72)	14.016	<0.001
	>18	1907(94.97)	338(17.72)		
	Missing	15(0.75)	3(20.00)		
Age of first sex	≤18	88(4.38)	28(31.82)	10.952	0.001
	>18	1902(94.72)	339(17.82)		
	Missing	18(0.90)	3(16.67)		
Age at first delivery	≤18	36(1.79)	13(36.84)	7.658	0.006
	>18	1942(96.81)	351(17.26)		
	Missing	28(1.40)	6(21.43)		
Number of pregnancy	≤1	340(16.93)	25(7.35)	34.805	<0.001
	2	909(45.27)	179(19.69)		
	≥3	743(37.00)	163(21.94)		
	Missing	16(0.80)	3(18.75)		
Number of deliveries	≤1	867(43.18)	110(12.69)	41.604	<0.001
	2	924(46.02)	197(21.32)		
	≥3	201(10.01)	60(29.85)		
	Missing	16(0.80)	3(18.75)		
Sexual status	Yes	1404(69.92)	256(18.23)	3.571	0.059
	No	377(18.77)	85(22.55)		
	Missing	227(11.30)	29(12.78)		
Condom	Yes	223(13.67)	29(13.00)	4.767	0.029
	No	1164(71.37)	223(19.16)		
	Missing	244(14.96)	33(13.52)		
Tubal ligation	Yes	315(19.31)	97(30.79)	43.693	<0.001
	No	1072(65.73)	155(14.46)		
	Missing	244(14.96)	33(13.52)		
Other contraceptive methods	Yes	84(5.15)	26(30.95)	9.828	0.002
	No	1303(79.89)	226(17.34)		
	Missing	244(14.96)	33(13.52)		
Wash vulva before sex	Always	756(46.35)	105(13.89)	16.124	<0.001
	Occasionally	386(23.67)	80(20.73)		
	Never	178(10.91)	44(24.72)		
	Missing	311(19.07)	56(18.01)		
Wash vulva after sex	Always	769(47.15)	106(13.78)	19.696	<0.001
	Occasionally	402(24.65)	82(20.40)		
	Never	155(9.50)	42(27.10)		
	Missing	305(18.70)	55(18.03)		
Spouse or sexual partner information					
Educational level	Below high school	1177(59.93)	257(21.84)	29.905	<0.001
	High school and above	690(35.13)	81(11.74)		
	Missing	97(4.94)	23(23.71)		
Employment status	Unemployed	128(6.52)	225(19.53)	383.675	<0.001
	Part-time employment	138(7.03)	18(13.04)		
	Full-time employment	536(27.29)	67(12.50)		
	Farmer	555(28.26)	134(24.14)		
	Retired	108(5.50)	21(19.44)		
	Others	387(19.70)	73(18.86)		
	Missing	112(5.70)	23(20.54)		

**Table 6:** Univariate logistic regression analysis of the perimenopausal group (40-59 years old) (P<0.1). HR-HPV: high-risk human papillomavirus; CNY: Chinese Yuan

	Characteristics	Classification	No. 0f cases (%^a^)	No. of HR-HPV+ (%^b^)	χ2	P-value
Profession		Farmer	745(45.29)	149(20)	20.683	0.002
		Individual business	102(6.20)	15(14.71)		
		Public servant	121(7.36)	7(5.79)		
		Worker	155(9.42)	18(11.61)		
		Retired	161(9.79)	23(14.29)		
		Others	262(15.93)	41(15.65)		
		Unemployed	94(5.71)	14(14.89)		
		Missing	5(0.30)	0(0.00)		
Educational level		Below junior high school	652(39.64)	134(20.55)	14.673	<0.001
		Junior high school and above	984(59.82)	132(13.41)		
		Missing	9(0.55)	1(11.11)		
Native place		Lueyang	1486(90.33)	252(16.96)	3.408	0.065
		Others	138(8.39)	15(10.87)		
		Missing	21(1.28)	0(0.00)		
Number of family members		1-3	828(50.33)	120(14.49)	4.146	0.042
		≥4	807(49.06)	147(18.22)		
		Missing	10(0.61)	0(0.00)		
Personal annual income		＜3000 CNY	322(19.57)	65(20.19)	5.582	0.018
		≥3000 CNY	1238(75.26)	183(14.78)		
		Missing	85(5.17)	19(22.35)		
Medical insurance		New rural cooperative medical care	951(57.81)	182(19.14)	13.162	0.001
		Urban medical insurance	489(29.73)	60(12.27)		
		Others	98(5.96)	11(11.22)		
		Missing	107(6.50)	14(13.08)		
Living environment and Living conditions		Excellent or good	1144(69.54)	167(14.60)	7.319	0.007
		General or poor	485(29.48)	97(20.00)		
		Missing	16(0.97)	3(18.75)		
Smoking		Present or past	18(1.09)	6(33.33)	-	0.058(Fisher)
		Never	1625(98.78)	261(16.06)		
		Missing	2(0.12)	0(0.00)		
History of lower reproductive tract disease		No	1143(69.48)	164(14.35)	9.765	0.002
		Yes	502(30.52)	103(20.52)		
History of HPV vaccination		Yes	19(1.16)	0(0.00)	-	0.058(Fisher)
		No	1593(96.84)	262(16.45)		
		Missing	33(2.01)	5(15.15)		
Do you know about HPV?		Yes	465(28.27)	58(12.47)	6.674	0.010
		No	1170(71.12)	207(17.69)		
		Missing	10(0.61)	2(20.00)		
Age of menarche		＜12	33(2.01)	6(18.18)	5.947	0.051
		12-14	1080(65.65)	158(14.63)		
		>14	521(31.67)	101(19.39)		
		Missing	11(0.67)	2(18.18)		
Menopausal		Yes	752(45.71)	150(19.95)	14.898	<0.001
		No	829(50.40)	106(12.79)		
		Missing	64(3.89)	11(17.19)		
Age of first marriage		≤18	55(3.34)	15(27.27)	5.105	0.024
		>18	1584(96.29)	251(15.85)		
		Missing	6(0.36)	1(16.67)		
Age of first sex		≤18	58(3.53)	15(25.86)	4.062	0.044
		>18	1577(95.87)	251(15.92)		
		Missing	10(0.61)	1(10.00)		
Age at first delivery		≤18	24(1.46)	7(29.17)	-	0.095 (Fisher)
		>18	1603(97.45)	258(16.09)		
		Missing	16(0.97)	2(12.50)		
Number of pregnancy		≤1	321(19.51)	22(6.85)	26.122	<0.001
		2	761(46.26)	138(18.13)		
		≥3	555(33.74)	106(19.10)		
		Missing	8(0.49)	1(12.50)		
Number of deliveries		0-1	834(50.70)	104(12.47)	18.411	<0.001
		2	716(43.53)	142(19.83)		
		≥3	87(5.29)	20(22.99)		
		Missing	8(0.49)	1(12.50)		
Sexual status		Yes	1290(78.42)	206(15.97)	3.922	0.048
		No	220(13.37)	47(21.36)		
		Missing	135(8.21)	14(10.37)		
Tubal ligation		Yes	271(19.02)	72(26.57)	28.542	<0.001
		No	1010(70.88)	133(13.17)		
		Missing	144(10.11)	15(10.42)		
Other contraceptive methods		Yes	73(5.12)	19(26.03)	5.787	0.016
		No	1208(84.77)	186(15.40)		
		Missing	144(10.11)	15(10.42)		
Wash vulva before sex		always	728(51.09)	95(13.05)	8.760	0.013
		Occasionally	346(24.28)	60(17.34)		
		Never	152(10.67)	33(21.71)		
		Missing	199(13.96)	32(16.08)		
Wash vulva after sex		always	740(51.93)	95(12.84)	11.577	0.003
		Occasionally	360(25.26)	62(17.22)		
		Never	131(9.19)	31(23.66)		
		Missing	194(13.61)	32(16.49)		
Spouse or sexual partner information						
Educational level		Below high school	918(57.05)	177(19.28)	18.219	<0.001
		High school and above	641(39.84)	72(11.23)		
		Missing	50(3.11)	10(20.00)		
Employment status		Unemployed	91(5.66)	16(17.58)	11.433	0.043
		Part-time employment	132(8.20)	18(13.64)		
		Full-time employment	525(32.63)	64(12.19)		
		Farmer	361(22.44)	70(19.39)		
		Retired	71(4.41)	14(19.72)		
		Others	369(22.93)	67(18.16)		
		Missing	60(3.73)	10(16.67)		
Smoking		Present or past	865(53.76)	150(17.34)	1056.773	<0.001
		Never	691(42.95)	97(14.04)		
		Missing	53(3.29)	12(22.64)		

**Table 7:** Univariate logistic regression analysis of the elderly group (60-65 years old) (P<0.1). HR-HPV: high-risk human papillomavirus

Characteristics	Classification	No. 0f cases (%^a^)	No. of HR-HPV+ (%^b^)	χ2	P-value
Educational level	Below junior high school	287(79.06)	88(30.66)	5.471	0.019
	Junior high school and above	67(18.46)	11(16.42)		
	Missing	9(2.48)	4(44.44)		
Living environment and living conditions	Excellent or good	208(57.3)	48(23.08)	4.513	0.034
	General or poor	144(39.67)	48(33.33)		
	Missing	11(3.03)	7(63.64)		
Age of menarche	＜12	2(0.55)	1(50.00)	5.508	0.064
	12-14	165(45.45)	56(33.94)		
	>14	186(51.24)	43(23.12)		
	Missing	10(2.75)	3(30.00)		
Age of first marriage	≤18	31(8.54)	14(45.16)	4.608	0.032
	>18	323(88.98)	87(26.93)		
	Missing	9(2.48)	2(22.22)		
Age of first sex	≤18	30(8.26)	13(43.33)	3.566	0.059
	>18	325(89.53)	88(27.08)		
	Missing	8(2.20)	2(25.00)		
Sexual status	Yes	114(31.4)	50(43.86)	11.637	0.001
	No	157(43.25)	38(24.20)		
	Missing	92(25.34)	15(16.3)		
Contraceptive method: tubal ligation	Yes	44(21.36)	25(56.82)	4.746	0.029
	No	62(30.10)	22(35.48)		
	Missing	100(48.54)	18(18.00)		
Spouse or sexual partner information					
Educational level	Below high school	259(72.96)	80(30.89)	3.144	0.076
	High school and above	49(13.80)	9(18.37)		
	Missing	49(13.80)	13(27.66)		

**Table 8:** Univariate logistic regression analysis of the overall group (40-65 years old) (P≥0.10). HR-HPV: high-risk human papillomavirus; CNY: Chinese Yuan

Characteristics	Classification	No. 0f cases (%^a^)	No. of HR-HPV+ (%^b^)	χ2	P-value
BMI	＜18.5	33(1.64)	4(12.12)	0.898	0.638
	18.5-23.9	978(48.71)	181(18.51)		
	≥24	967(48.16)	180(18.61)		
	Missing	30(1.49)	5(16.67)		
Ethnicity	Han	1975(98.36)	366(18.53)	-	1.000(Fisher)
	Others	16(0.80)	3(18.75)		
	Missing	17(0.85)	1(5.88)		
Religion	Yes	31(1.54)	7(22.58)	0.372	0.542
	No	1950(97.11)	357(18.31)		
	Missing	27(1.34)	6(22.22)		
Annual household income	＜30000 CNY	1163(57.92)	219(18.83)	1.769	0.184
	≥30000 CNY	719(35.81)	118(16.41)		
	Missing	126(6.27)	33(26.19)		
Alcohol consumption	Present or past	65(3.24)	15(23.08)	0.956	0.328
	Never	1935(96.36)	354(18.29)		
	Missing	8(0.40)	1(12.50)		
History of pelvic infection	No	1483(73.85)	267(18.00)	0.673	0.412
	Yes	525(26.15)	103(19.62)		
History of adenomyosis	No	1886(93.92)	343(18.19)	1.186	0.276
	Yes	122(6.08)	27(22.13)		
History of endometriosis	No	2000(99.6)	370(18.5)	-	0.365(Fisher)
	Yes	8(0.40)	0(0.00)		
History of abnormal fallopian tubes	No	2003(99.75)	370(18.47)	-	0.592(Fisher)
	Yes	5(0.25)	0(0.00)		
History of ovarian abnormalities	No	1986(98.9)	367(18.48)	-	0.783(Fisher)
	Yes	22(1.10)	3(13.64)		
History of annexitis	No	2000(99.6)	368(18.4)	-	0.645(Fisher)
	Yes	8(0.40)	2(25.00)		
History of uterine fibroids	No	1985(98.85)	364(18.34)	-	0.413(Fisher)
	Yes	23(1.15)	6(26.09)		
History of blood transfusion and donation	Yes	253(12.6)	38(15.02)	2.311	0.128
	No	1711(85.21)	325(18.99)		
	Missing	44(2.19)	7(15.91)		
Allergic history	Yes	237(11.8)	38(16.03)	1.097	0.295
	No	1740(86.65)	328(18.85)		
	Missing	31(1.54)	4(12.90)		
Regular cervical cancer screening	Yes	488(24.30)	80(16.39)	1.864	0.172
	No	1493(74.35)	286(19.16)		
	Missing	27(1.34)	4(14.81)		
Regular gynecological Examinations	Yes	566(28.19)	105(18.55)	0.006	0.940
	No	1262(62.85)	236(18.7)		
	Missing	180(8.96)	29(16.11)		
Age of menarche	＜12	35(1.74)	7(20.00)	3.102	0.212
	12-14	1245(62)	214(17.19)		
	>14	707(35.21)	144(20.37)		
	Missing	21(1.05)	5(23.81)		
Age at menopause	<50	544(46.3)	134(24.63)	1.748	0.186
	≥50	541(46.04)	115(21.26)		
	Missing	90(7.66)	15(16.67)		
Delivery history	Yes	2006(99.90)	370(18.44)	-	1.000(Fisher)
	No	2(0.10)	0(0.00)		
Number of abortions	0	1092(54.38)	205(18.77)	0.392	0.822
	1	542(26.99)	95(17.53)		
	≥2	359(17.88)	67(18.66)		
	Missing	15(0.75)	3(20.00)		
Number of marriages	≤1	1923(95.77)	358(18.62)	0.221	0.638
	≥2	50(2.49)	8(16.00)		
	Missing	35(1.74)	4(11.43)		
Family history of cancer	Yes	166(8.27)	34(20.48)	0.492	0.483
	No	1822(90.74)	333(18.28)		
	Missing	20(1.00)	3(15.00)		
Family history of cervical cancer	Yes	63(3.14)	13(20.63)	0.204	0.651
	No	1925(95.87)	354(18.39)		
	Missing	20(1.00)	3(15.00)		
Number of sexual partners in a lifetime	1	1945(96.86)	358(18.41)	0.170	0.680
	≥2	38(1.89)	6(15.79)		
	Missing	25(1.25)	6(24.00)		
Frequency of sexual activity	Less than once a month	133(8.15)	17(12.78)	3.906	0.142
	Once to four times a month	973(59.66)	167(17.16)		
	At least 5 times a month	194(11.89)	24(12.37)		
	Missing	331(20.29)	77(23.26)		
IUD	Yes	299(18.33)	59(19.73)	0.627	0.429
	No	1088(66.71)	193(17.74)		
	Missing	244(14.96)	33(13.52)		
OC	Yes	12(0.74)	0(0.00)	-	0.139(Fisher)
	No	1375(84.3)	252(18.33)		
	Missing	244(14.96)	33(13.52)		
Bathing frequency	At least once every 5 days	1299(64.69)	229(17.63)	1.153	0.283
	Once more than 5 days	684(34.06)	134(19.59)		
	Missing	25(1.25)	7(28.00)		
Change underwear frequency	Once every 1 - 3 days	1635(81.42)	289(17.68)	1.128	0.288
	Once more than 3 days	338(16.83)	68(20.12)		
	Missing	35(1.74)	13(37.14)		
Spouse or sexual partner information					
Spouse or sexual partner	Yes	1964(97.81)	361(18.38)	0.123	0.726
	No	44(2.19)	9(20.45)		
Number of marriages	≤1	1826(92.97)	331(18.13)	0.259	0.611
	≥2	40(2.04)	6(15.00)		
	Missing	98(4.99)	24(24.49)		
History of circumcision	Yes	37(1.88)	3(8.11)	2.658	0.103
	No	1742(88.7)	324(18.6)		
	Missing	185(9.42)	34(18.38)		
Smoking	Present or past	1037(52.8)	199(19.19)	2.531	0.112
	Never	826(42.06)	135(16.34)		
	Missing	101(5.14)	27(26.73)		
Alcohol consumption	Present or past	958(48.78)	164(17.12)	1.050	0.306
	Never	908(46.23)	172(18.94)		
	Missing	98(4.99)	25(25.51)		

**Table 9:** Univariate logistic regression analysis of the perimenopausal group (40-59 years old) (P≥0.10). HR-HPV: high-risk human papillomavirus; CNY: Chinese Yuan

Characteristics	Classification	No. 0f cases (%^a^)	No. of HR-HPV+ (%^b^)	χ2	P-value
BMI	＜18.5	24(1.46)	2（8.33)	1.217	0.544
	18.5-23.9	830(50.46)	134（16.14)		
	≥24	767(46.63)	128（16.69)		
	Missing	24(1.46)	3（12.50)		
Ethnicity	Han	1622(98.6)	265（16.34)	-	1.000（Fisher）
	Others	13(0.79)	2（15.38)		
	Missing	10(0.61)	0（0.00)		
Religion	Yes	19(1.16)	5（26.32)	-	0.218（Fisher）
	No	1610(97.87)	260（16.15)		
	Missing	16(0.97)	2（12.50)		
Annual household income	＜30000 CNY	906(55.08)	148（16.34)	0.255	0.613
	≥30000 CNY	650(39.51)	100（15.38)		
	Missing	89(5.41)	19（21.35)		
Alcohol consumption	Present or past	55(3.34)	12（21.82)	1.286	0.257
	Never	1586(96.41)	255（16.08)		
	Missing	4(0.24)	0（0.00)		
History of pelvic infection	No	1210(73.56)	189（15.62)	1.257	0.262
	Yes	435(26.44)	78（17.93)		
History of adenomyosis	No	1537(93.43)	245（15.94)	1.457	0.228
	Yes	108(6.57)	22（20.37)		
History of endometriosis	No	1639(99.64)	267（16.29)	-	0.598(Fisher)
	Yes	6(0.36)	0（0.00)		
History of abnormal fallopian tubes	No	1641(99.76)	267（16.27)	-	1.000(Fisher)
	Yes	4(0.24)	0（0.00)		
History of ovarian abnormalities	No	1627(98.91)	266（16.35)	-	0.338(Fisher)
	Yes	18(1.09)	1（5.56)		
History of annexitis	No	1638(99.57)	265（16.18)	-	0.318(Fisher)
	Yes	7(0.43)	2（28.57)		
History of uterine fibroids	No	1623(98.66)	262（16.14)	-	0.384(Fisher)
	Yes	22(1.34)	5（22.73)		
History of blood transfusion and donation	Yes	235(14.29)	33（14.04)	1.095	0.295
	No	1377(83.71)	231（16.78)		
	Missing	33(2.01)	3（9.09)		
Allergic history	Yes	193(11.73)	29（15.03)	0.315	0.575
	No	1432(87.05)	238（16.62)		
	Missing	20(1.22)	0（0.00)		
Regular cervical cancer screening	Yes	402(24.44)	56（13.93)	2.284	0.131
	No	1225(74.47)	210（17.14)		
	Missing	18(1.09)	1（5.56)		
Regular gynecological examinations	Yes	476(28.94)	75（15.76)	0.226	0.635
	No	1034(62.86)	173（16.73)		
	Missing	135(8.21)	19（14.07)		
Age at menopause	<50	393(48.16)	86（21.88)	1.741	0.187
	≥50	350(42.89)	63（18.00)		
	Missing	73(8.95)	12（16.44)		
Number of abortions	0	851(51.73)	131（15.39)	1.441	0.487
	1	470(28.57)	77（16.38)		
	≥2	317(19.27)	58（18.30)		
	Missing	7(0.43)	1（14.29)		
Number of marriages	≤1	1573(95.62)	260（16.53)	0.833	0.361
	≥2	44(2.67)	5（11.36)		
	Missing	28(1.70)	2（7.14)		
Family history of cancer	Yes	148(9.00)	27（18.24)	0.461	0.497
	No	1486(90.33)	239（16.08)		
	Missing	11(0.67)	1（9.09)		
Family history of cervical cancer	Yes	57(3.47)	12（21.05)	0.988	0.320
	No	1577(95.87)	254（16.11)		
	Missing	11(0.67)	1（9.09)		
Number of sexual partners in a lifetime	1	1595(96.96)	261（16.36)	2.204	0.138
	≥2	31(1.88)	2（6.45)		
	Missing	19(1.16)	4（21.05)		
Frequency of sexual activity	Less than once a month	110（7.72)	12（10.91)	4.509	0.105
	Once to four times a month	938（65.82)	154（16.42)		
	At least 5 times a month	189（13.26)	22（11.64)		
	Missing	188（13.19)	32（17.02)		
IUD	Yes	287（20.14)	54（18.82)	2.176	0.140
	No	994（69.75)	151（15.19)		
	Missing	144（10.11)	15（10.42)		
OC	Yes	12（0.84)	0（0.00)	-	0.233(Fisher)
	No	1269（89.05)	205（16.15)		
	Missing	144（10.11)	15（10.42)		
Condom	Yes	215（15.09)	27（12.56)	2.281	0.131
	No	1066（74.81)	178（16.7)		
	Missing	144（10.11)	15（10.42)		
Bathing frequency	At least once every 5 days	1112(67.6)	173（15.56)	1.187	0.276
	Once more than 5 days	520(31.61)	92（17.69)		
	Missing	13(0.79)	2（15.38)		
Change underwear frequency	Once every 1 to 3 days	1439(87.48)	229（15.91)	0.127	0.722
	Once more than 3 days	183(11.12)	31（16.94)		
	Missing	23(1.40)	7（30.43)		
Spouse or sexual partner information					
Spouse or sexual partner	Yes	1609(97.81)	259（16.10)	0.972	0.324
	No	36(2.19)	8（22.22)		
Number of marriages	≤1	1525（94.78)	245（16.07)	0.374	0.541
	≥2	33（2.05)	4（12.12)		
	Missing	51（3.17)	10（19.61)		
History of circumcision	Yes	32（1.99)	2（6.25)	2.404	0.121
	No	1463（90.93)	241（16.47)		
	Missing	114（7.09)	16（14.04)		
Alcohol consumption	Present or past	838（52.08)	131（15.63)	0.156	0.693
	Never	721（44.81)	118（16.37)		
	Missing	50（3.11)	10（20.00)		

**Table 10:** Univariate logistic regression analysis of the elderly group (60-65 years old) (P≥0.10). HR-HPV: high-risk human papillomavirus; CNY: Chinese Yuan

Characteristics	Classification	No. 0f cases (%^a^)	No. of HR-HPV+ (%^b^)	χ2	P-value
BMI	＜18.5	9(2.48)	2(22.22)	1.557	0.459
	18.5-23.9	148(40.77)	47(31.76)		
	≥24	200(55.1)	52(26.00)		
	Missing	6(1.65)	2(33.33)		
Profession	Farmer	317(87.33)	94(29.65)	-	0.759（Fisher）
	Individual business	3(0.83)	0(0.00)		
	Public servant	0(0.00)	0(0.00)		
	Worker	0(0.00)	0(0.00)		
	Retired	21(5.79)	4(19.05)		
	Others	8(2.20)	2(25.00)		
	Unemployed	10(2.75)	2(20.00)		
	Missing	4(1.10)	1(25.00)		
Native place	Lueyang	345(95.04)	97(28.12)	-	0.514(Fisher)
	Others	11(3.03)	4(36.36)		
	Missing	7(1.93)	2(28.57)		
Ethnicity	Han	353(97.25)	101(28.61)	-	1.000(Fisher)
	Others	3(0.83)	1(33.33)		
	Missing	7(1.93)	1(14.29)		
Religion	Yes	12(3.31)	2(16.67)	-	0.521(Fisher)
	No	340(93.66)	97(16.67)		
	Missing	11(3.03)	4(36.36)		
Number of family members	1-3	115(31.68)	35(30.43)	0.482	0.482
	≥4	242(66.67)	65(26.86)		
	Missing	6(1.65)	3(50.00)		
Personal annual income	＜3000 CNY	136(37.47)	31(22.79)	1.052	0.305
	≥3000 CNY	183(50.41)	51(27.87)		
	Missing	44(12.12)	21(47.73)		
Annual household income	＜30000 CNY	257(70.80)	71(27.63)	0.065	0.799
	≥30000 CNY	69(19.01)	18(26.09)		
	Missing	37(10.19)	14(37.84)		
Medical insurance	New rural cooperative medical care	285(78.51)	88(30.88)	-	0.243(Fisher)
	Urban medical insurance	27(7.44)	5(18.52)		
	Others	4(1.10)	0(0.00)		
	Missing	47(12.95)	10(21.28)		
Smoking	Present or past	10(2.75)	5(50.00)	-	0.155 (Fisher)
	Never	349(96.14)	97(27.79)		
	Missing	4(1.10)	1(25.00)		
Alcohol consumption	Present or past	10(2.75)	3(30.00)	-	1.000(Fisher)
	Never	349(96.14)	99(28.37)		
	Missing	4(1.10)	1(25.00)		
History of pelvic infection	No	273(75.21)	78(28.57)	0.021	0.885
	Yes	90(24.79)	25(27.78)		
History of adenomyosis	No	349(96.14)	98(28.08)	0.386	0.534
	Yes	14(3.86)	5(35.71)		
History of endometriosis	No	361(99.45)	103(28.53)	-	1.000(Fisher)
	Yes	2(0.55)	0(0.00)		
History of abnormal fallopian tubes	No	362(99.72)	103(28.45)	-	1.000(Fisher)
	Yes	1(0.28)	0(0.00)		
History of ovarian abnormalities	No	359(98.9)	101(28.13)	-	0.319(Fisher)
	Yes	4(1.10)	2(50.00)		
History of lower reproductive tract disease	No	264(72.73)	74(28.03)	0.057	0.812
	Yes	99(27.27)	29(29.29)		
History of annexitis	No	362(99.72)	103(28.45)	-	1.000(Fisher)
	Yes	1(0.28)	0(0.00)		
History of uterine fibroids	No	362(99.72)	102(28.18)	-	0.284(Fisher)
	Yes	1(0.28)	1(100.00)		
History of blood transfusion and donation	Yes	18(4.96)	5(27.78)	0.001	0.973
	No	334(92.01)	94(28.14)		
	Missing	11(3.03)	4(36.36)		
Allergic history	Yes	44(12.12)	9(20.45)	1.464	0.226
	No	308(84.85)	90(29.22)		
	Missing	11(3.03)	4(36.36)		
Regular cervical cancer screening	Yes	86(23.69)	24(27.91)	0.007	0.936
	No	268(73.83)	76(28.36)		
	Missing	9(2.48)	3(33.33)		
Regular gynecological examinations	Yes	90(24.79)	30(33.33)	1.014	0.314
	No	228(62.81)	63(27.63)		
	Missing	45(12.4)	10(22.22)		
History of hpv vaccination	Yes	1(0.28)	0(0.00)	-	1.000(Fisher)
	No	349(96.14)	102(29.23)		
	Missing	13(3.58)	1(7.69)		
Do you know about HPV?	Yes	23(6.34)	8(34.78)	0.545	0.460
	No	333(91.74)	92(27.63)		
	Missing	7(1.93)	3(42.86)		
Menopausal	Yes	351(96.69)	101(28.77)	-	0.581(Fisher)
	No	4(1.10)	0(0.00)		
	Missing	8(2.20)	2(25.00)		
Age at menopause	<50	151(42.06)	48(31.79)	0.849	0.357
	≥50	191(53.2)	52(27.23)		
	Missing	17(4.74)	3(17.65)		
Number of pregnancy	≤1	19(5.23)	3(15.79)	1.859	0.395
	2	148(40.77)	41(27.70)		
	≥3	188(51.79)	57(30.32)		
	Missing	8(2.20)	2(25.00)		
Number of abortions	0	241(66.39)	74(30.71)	2.040	0.361
	1	72(19.83)	18(25.00)		
	≥2	42(11.57)	9(21.43)		
	Missing	8(2.20)	2(25.00)		
Number of marriages	≤1	350(96.42)	98(28.00)	-	0.358(Fisher)
	≥2	6(1.65)	3(50.00)		
	Missing	7(1.93)	2(28.57)		
Family history of cancer	Yes	18(4.96)	7(38.89)	0.998	0.318
	No	336(92.56)	94(27.98)		
	Missing	9(2.48)	2(22.22)		
Family history of cervical cancer	Yes	6(1.65)	1(16.67)	-	0.679(Fisher)
	No	348(95.87)	100(28.74)		
	Missing	9(2.48)	2(22.22)		
Age at first delivery	≤18	12(3.31)	6(50.00)	-	0.105(Fisher)
	>18	339(93.39)	93(27.43)		
	Missing	12(3.31)	4(33.33)		
Number of deliveries	0-1	33(9.09)	6(18.18)	4.589	0.101
	2	208(57.30)	55(26.44)		
	≥3	114(31.40)	40(35.09)		
	Missing	8(2.20)	2(25.00)		
Number of sexual partners in a lifetime	1	350(96.42)	97(27.71)	-	0.103(Fisher)
	≥2	7(1.93)	4(57.14)		
	Missing	6(1.65)	2(33.33)		
Frequency of sexual activity	Less than once a month	23(11.17)	5(21.74)	-	0.490(Fisher)
	Once to four times a month	35(16.99)	13(37.14)		
	At least 5 times a month	5(2.43)	2(40.00)		
	Missing	143(69.42)	45(31.47)		
Condom	Yes	8(3.88)	2(25.00)	-	0.296(Fisher)
	No	98(47.57)	45(45.92)		
	Missing	100(48.54)	18(18.00)		
IUD	Yes	12(5.83)	5(41.67)	0.039	0.843
	No	94(45.63)	42(44.68)		
	Missing	100(48.54)	18(18.00)		
Other contraceptive methods	Yes	11(5.34)	7(63.64)	-	0.210(Fisher)
	No	95(46.12)	40(42.11)		
	Missing	100(48.54)	18(18.00)		
Wash vulva before sex	Always	28(13.59)	10(35.71)	1.392	0.499
	Occasionally	40(19.42)	20(50.00)		
	Never	26(12.62)	11(42.31)		
	Missing	112(54.37)	24(21.43)		
Wash vulva after sex	Always	29(14.08)	11(37.93)	0.687	0.709
	Occasionally	42(20.39)	20(47.62)		
	Never	24(11.65)	11(45.83)		
	Missing	111(53.88)	23(20.72)		
Bathing frequency	At least once every 5 days	187(51.52)	56(29.95)	0.817	0.366
	Once more than 5 days	164(45.18)	42(25.61)		
	Missing	12(3.31)	5(41.67)		
Change underwear frequency	Once every 1 - 3 days	196(53.99)	60(30.61)	1.967	0.161
	Once more than 3 days	155(42.70)	37(23.87)		
	Missing	12(3.31)	6(50.00)		
Spouse or sexual partner information					
Spouse or sexual partner	Yes	355(97.80)	102(28.73)	-	0.449(Fisher)
	No	8(17.78)	1(12.50)		
Employment status	Unemployed	37(10.42)	9(24.32)	-	0.310(Fisher)
	Part-time employment	6(1.69)	0(0.00)		
	Full-time employment	11(3.10)	3(27.27)		
	Farmer	194(54.65)	64(32.99)		
	Retired	37(10.42)	7(18.92)		
	Others	18(5.07)	6(33.33)		
	Missing	52(14.65)	13(25.00)		
Number of marriages	≤1	301(84.79)	86(28.57)	-	1.000(Fisher)
	≥2	7(1.97)	2(28.57)		
	Missing	47(13.24)	14(29.79)		
History of circumcision	Yes	5(1.41)	1(20.00)	-	1.000(Fisher)
	No	279(78.59)	83(29.75)		
	Missing	71(20.00)	18(25.35)		
Smoking	Present or past	172(48.45)	49(28.49)	-	1.000(Fisher)
	Never	135(38.03)	38(28.15)		
	Missing	48(13.52)	15(31.25)		
Alcohol consumption	Present or past	120(33.8)	33(27.5)	0.068	0.794
	Never	187(52.68)	54(28.88)		
	Missing	48(13.52)	15(31.25)		

**Table 11:** Multiple interpolation methods for three groups.

Age groups	Seed	Number of imputations	Cronbach alpha coefficient	χ2 (Hosmer-Lemeshow test)	P-value	Correct prediction rate(%)
40-65	20 000 000	5	0.416	13.313	0.102	81.8
40-59	200 000 000	5	0.313	12.299	0.138	83.8
60-65	20 000 000	5	0.253	3.174	0.787	72.7

**Table 12:** Results of multivariate logistic regression analysis on factors influencing HR-HPV infection in the three groups without multiple imputation. HR-HPV: High-risk Human Papillomavirus.

Characteristics	Classification	SE	P-value	OR(95% CI)
The overall group				
Number of pregnancy	-	-	0.020	-
	≤1	-	-	1（Reference）
	2	0.266	0.006	2.07 [1.23-3.49]
	≥3	0.27	0.009	2.02[1.19-3.44]
History of lower reproductive tract disease	No	-	-	1（Reference）
	Yes	0.15	0.036	1.37[1.02-1.84]
Contraceptive method: tubal ligation	Yes	-	-	1（Reference）
	No	0.2	0.002	0.53[0.36-0.79]
Other contraceptive methods	Yes	-	-	1（Reference）
	No	0.343	0.046	0.51[0.26-0.99]
Wash vulva before sex	-	-	0.036	-
	Frequently	-	-	1（Reference）
	Occasionally	0.196	0.234	1.26[0.86-1.85]
	Never	0.249	0.011	1.89[1.16-3.07]
The perimenopausal group				
Number of pregnancy	-	-	0.033	-
	≤1	-	-	1（Reference）
	2	0.302	<0.01	2.18[1.21-3.95]
	≥3	0.31	0.02	2.05[1.12-3.77]
Contraceptive method: tubal ligation	Yes	-	-	1（Reference）
	No	0.18	<0.001	0.49[0.35-0.70]
The elderly group				
Educational level	Below junior high school	-	-	1（Reference）
	Junior high school and above	0.44	0.037	0.40[0.17-0.95]
Sexual status	Yes	-	-	1（Reference）
	No	0.399	<0.001	0.27[0.13-0.60]
