# Correction: Prevalence of High-Risk Human Papillomavirus Infection, Associated Risk Factors, and Relationship With Cervical Precancerous Lesions in Perimenopausal and Older Women in an Area With High Cervical Cancer Incidence in China

**DOI:** 10.7759/cureus.c171

**Published:** 2024-04-30

**Authors:** Ruoyi Zhang, Wei Xu, Siyuan Yang, Dehua Hu, Li Bai, Rumei Xiang, Xiaowei Zhao, Yuxian Nie, Qiu-ling Shi

**Affiliations:** 1 Epidemiology and Health Statistics, School of Public Health, Chongqing Medical University, Chongqing, CHN; 2 Nursing, School of Nursing, Chongqing Medical University, Chongqing, CHN; 3 Obstetrics and Gynecology, Centre of Lueyang Maternal and Child Health Care Hospital, Shaanxi, CHN; 4 Epidemiology and Health Statistics, College of Public Health, Chongqing Medical University, Chongqing, CHN; 5 Biomedical Engineering, State Key Laboratory of Ultrasound in Medicine and Engineering, Chongqing Medical University, Chongqing, CHN; 6 Epidemiology and Health Statistics, College of Public Health, State Key Laboratory of Ultrasound in Medicine and Engineering, Chongqing Medical University, Chongqing, CHN

This article has been corrected to disclose financial relationships omitted from the original published version.

The funding for this project was provided by:

Foundation of State Key Laboratory of Ultrasound in Medicine and Engineering (No. 2021KFKT007) Chongqing Social Science Planning Project (No. 2022BS081) China Postdoctoral Science Foundation Funded Project (No. 2022MD723732) Natural Science Foundation of Chongqing, China (No. 2023NSCQ-BHX0036)

The authors and journal deeply regret that this error was not identified and addressed prior to publication.

 

